# The effect of asparagine-13 in porcine epidemic diarrhea virus envelope protein on pathogenicity

**DOI:** 10.1186/s13567-025-01511-1

**Published:** 2025-04-19

**Authors:** Zhiwei Li, Zhiqian Ma, Xiaojing Zhao, Yongqi Li, Congsen Zheng, Yang Li, Xuyang Guo, Lele Xu, Zifang Zheng, Haixue Zheng, Shuqi Xiao

**Affiliations:** State Key Laboratory for Animal Disease Control and Prevention, Lanzhou Veterinary Research Institute, Chinese Academy of Agricultural Sciences, College of Veterinary Medicine, Lanzhou University, Lanzhou, 730000 Gansu China

**Keywords:** Porcine epidemic diarrhea virus, virulence factor, envelope protein, pro-inflammatory cytokines, reverse genetic system

## Abstract

The pathogenesis of porcine epidemic diarrhea virus (PEDV) has not been fully clarified, which seriously hinders the prevention of the disease. The envelope (E) protein of PEDV induces the expression of pro-inflammatory cytokines, but the role of these inflammatory reactions in PEDV pathogenicity is still unknown. In this study, the asparagine at position 13 was found to be crucial to PEDV E protein induced inflammatory response. Exogenously expressing the parent E protein, rather than the E mutant carrying N13A, induces the activation of NF-κB and expression of inflammatory factors, including IL-6, IL-8, and TNF-α. Compared with the parental rPEDV strain, the recombinant strain rPEDV-E_N13A_ exhibited a significantly lower infectious titer and formed smaller plaques. In addition, rPEDV-E_N13A_ induced lower expression of inflammatory factors in vitro and in vivo. The pathogenicity assay shows that the rPEDV-E_N13A_ strain caused diminished fecal PEDV RNA shedding, delayed death time, and milder histopathological lesions to intestinal villi. Our data provide a unique perspective for exploring the pathogenic mechanism of PEDV and a new target for the development of attenuated PEDV live vaccines.

## Introduction

Porcine epidemic diarrhea virus (PEDV) results in porcine epidemic diarrhea (PED), causing diarrhea, vomiting, dehydration, and high mortality rates in neonatal piglets, which is not effectively controlled to date [1, 2]. Analyzing the pathogenic mechanism of PEDV is crucial for preventing and controlling the disease. PEDV is a single-stranded, positive-sense RNA virus with a genome size of around 28 kb. The PEDV genome consists of 4 structural proteins, 16 non-structural proteins (nsp1–nsp16), one accessory protein (ORF3), one 5′cap, and one 3′poly (A) tail [3]. Among them, the envelope (E) protein is an interferon antagonist while functioning as a pro-inflammatory factor [4–6].

Viral infections usually induce inflammatory responses associated with tissue damage, and some virus proteins have been identified as key proteins that induce inflammatory responses [7]. Pseudorabies virus (PRV) infection generates inflammatory damage, such as encephalitis, and interstitial pneumonia. The non-structural protein UL4 of PRV is responsible for apoptosis-associated speck-like protein containing a caspase-recruitment domain (ASC)-dependent inflammasome activation [8]. Proinflammatory cytokine production in coronavirus infections results in acute lung injuries and multiple organ dysfunction syndrome [9–11]. Recently, the E protein in severe coronaviruses, such as Severe acute respiratory syndrome coronavirus 2 (SARS-CoV-2), Severe acute respiratory syndrome coronavirus 1 (SARS-CoV-1), or Middle East respiratory syndrome coronavirus (MERS-CoV), has been found to trigger excessive inflammatory factor release by interaction with Transmembrane emp24 domain-containing protein 10 (TMED10) [12]. SARS-CoV-2 spike protein induces IL-18-mediated cardiopulmonary inflammation by inhibiting mitophagy and increasing mitochondrial reactive oxygenation species [13]. SARS-CoV-2 nucleocapsid (N) promotes NOD-like receptor protein 3 (NLRP3) inflammasome activation to induce hyperinflammation and severe symptoms of coronavirus disease-19 (COVID-19) by promoting the binding of NLRP3 with ASC and facilitating NLRP3 inflammasome assembly [14]. PEDV infections cause the expression of pro-inflammatory cytokines [15]. PEDV E protein induces proinflammatory cytokine production via the activation of NF-κB signaling pathway [4, 6]. Many PEDV proteins have been identified to affect the virulence of the virus through multiple pathways [16, 17]. However, the role of the pro-inflammatory effect of PEDV protein, including the E protein, in viral pathogenicity still needs to be elucidated.

In this study, the eukaryotic expression vector carrying N13A in the E gene and the recombinant strain rPEDV-E_N13A_ were constructed using the highly virulent rPEDV GII group strain as the backbone. Compared to the wild-type E protein (E), exogenously expressed E mutant carrying N13A (E_N13A_) induces lower expression of the inflammatory factors, including IL-8, IL-6, and TNF-α. Compared to the parental strain (rPEDV), the rPEDV-E_N13A_ strain also induced lower expression of the IL-8, IL-6, and TNF-α inflammatory factors in vitro and in vivo. The piglet pathogenicity shows that the rPEDV-E_N13A_ strain caused diminished fecal PEDV RNA shedding and delayed death time. Our data indicate that the 13th asparagine of E protein is a key site that triggers inflammatory response and affects viral virulence, which enriches the content of the pathogenic mechanism of PEDV and provides a new target for the development of attenuated live vaccines.

## Materials and methods

### Viruses, cells, antibodies, and plasmids

MARC-145 cells and HEK-293T cells were preserved in our laboratory [18]. MARC-145 cells and HEK-293T cells were cultured in Dulbecco modified Eagle medium (DMEM; Gibco, CA, USA) supplemented with 10% fetal bovine serum (FBS; TransGen Biotech, China), 100 U/mL penicillin, and 100 μg/mL streptomycin. The sample that contains the CH/SX/2016 strain (GenBank No. MT787025) was stored in our laboratory. PEDV HNXP strain was kindly provided by Dr Changxu Song. Our group constructed and successfully rescued the recombinant strains rCH/SX/2016-S_HNXP_ (rPEDV) [19]. The propagation of PEDV was performed in MARC-145 cells. PEDV N and M monoclonal antibodies were prepared and stored in our laboratory. The pCAGGS and the bacterial artificial chromosome (BAC) vector pBeloBAC11 used in this study were stored in our laboratories.

### Strategies for constructing and rescuing chimeric full-length cDNA clones of PEDV

The rPEDV-E_N13A_ strain was generated using a similar strategy as before with little modification [19]. In brief, the complete gene sequence of CH/SX/2016 strain was obtained by reverse transcription polymerase chain reaction (RT-PCR) and 5’- and 3’-RACE. The full-length rCH/SX/2016 was divided into nine continuous fragments (A: nucleotide [nt]1–41, B: nt 42–3459, C: nt 3460–7617, D: nt 7618–13008, E: nt 13009–16888, F: nt 16889–20763, G: nt 20764–24079, H: nt 24080–27184, and I: nt 27185–28067). All nine continuous fragments were subsequently cloned into the pBeloBAC11 vector to obtain the full-length CH/SX/2016 clone. A similar strategy was used to generate other full-length cDNA clones. MARC-145 cells were grown to 80% confluency in a six-well plate, and 2.5 μg of the recombinant BAC plasmids were transfected into MARC-145 cells using Lipofectamine 3000 transfection reagent. MARC-145 cells were cultured in DMEM. The cytopathic effect (CPE) was monitored daily after transfection. When CPE was obvious, the cells and supernatants were collected and freeze-thawed for propagation.

### Identification of the recombinant strains

MARC-145 cells were infected with rPEDV and rPEDV-E_N13A_ and collected at 36 hpi. The RNA of the samples were extracted using TRIzol reagent (TaKaRa, Japan) and reverse transcribed using HiScript II Q RT SuperMix for qPCR (Vazyme, China) according to the manufacturer’s instructions. For sequencing, the fragments were amplified using corresponding primers, which are listed in Table 1.

**Table 1:** **Primers in this study**

Primer name		Sequence (5′–3′)	Usage	Refs.
PEDV-nsp1-F		TTGTGGTCGGCACTACCAAG	RT-qPCR	[5]
PEDV-nsp1-R		CACGACGACCAAAAGTGAGC		
pIL-6-F		CTGGCAGAAAACAACCTGAACC		[34]
pIL-6-R		TGATTCTCATCAAGCAGGTCTCC		
pIL-8-F		CCGTGTCAACATGACTTCCAA		
pIL-8-R		GCCTCACAGAGAGCTGCAGAA		
pTNF-α-F		AACCTCAGATAAGCCCGTCG		
pTNF-α-R		ACCACCAGCTGGTTGTCTTT		
pGAPDH-F		CCTTCCGTGTCCCTACTGCCAAC		[35]
pGAPDH-R		GACGCCTGCTTCACCACCTTCT		
hTNF-α-F		GGCGTGGAGCTGAGAGATAAC		[36]
hTNF-α-R		GGTGTGGGTGAGGAGCACAT		
hIL-6-F		GTCAGGGGTGGTTATTGCAT		[37]
hIL-6-R		AGTGAGGAACAAGCCAGAGC		
hIL-8-F		TGTGAAGGTGCAGTTTTGCC		
hIL-8-R		CACCCAGTTTTCCTTGGGGT		
mTNF-α-F		TGTGTCTGCTGCACTTTGGAGTG		[38]
mTNF-α-R		TTGAGGGTTTGCTACAACATGG		
mIL-6-F		GCTGCAGGCGCAGAACCA		[39]
mIL-6-R		AAAGCTGCGCAGGATGAGA		
mIL-8-F		CTGGCGGTGGCTCTCTTG		
mIL-8-R		CCTTGGCAAAACTGCACCTT		
h/mβ-actin-F		ATCGTGCGTGACATTAAG		[40]
h/mβ-actin-R		ATTGCCAATGGTGATGAC		
PEDV-E-F		GACACAGTTGTCAAAGATGTC	Sequencing	[5]
PEDV-E-R		TTTGCGGCCACGATCCTGAAAA		
PEDV-N-F		GAATTCCCAAGGGCGAAAAT	RT-qPCR	[41]
N gene probe		FAM-CGTAGCAGCTTGCTTCGGACCCA-BHQ		
PEDV-N-R		TTTTCGACAAATTCCGCATCT		

### Indirect immunofluorescence assay (IFA)

IFA was performed as before with little modification [19]. MARC-145 cells were grown on a 12-well plate infected with rPEDV, rPEDV-E_N13A_, or mock-infected for 36 h. Cells were washed with PBS and fixed with 4% paraformaldehyde for 10 min at 37 °C, followed by membrane permeabilization with 0.25% Triton X-100 in phosphate buffered saline (PBS) for 10 min at 37 °C. Cells were blocked with 1% BSA at 37 °C for 30 min, then incubated with mouse anti-N/anti-M monoclonal antibody at a dilution of 1:1000 at 37 ℃ for 1 h. Cells were washed with PBS three times and incubated with Fluorescein (FITC)-AffiniPure Goat Anti-Mouse IgG (H+L) at 1:200 at 37 ℃ for 1 h. Cells were washed with PBS three times and stained with 4-6-diamidino-2-phenylindole (DAPI) for 10 min at room temperature. The images were taken with a fluorescence microscope.

### Western blot

MARC-145 cells were grown to 90% confluency in 6-well plates and infected with rPEDV and rPEDV-E_N13A_. Cells were harvested at 36 hpi. The cells were lysed with 200 μL of ice-cold RIPA buffer for 30 min on ice, and then the supernatant proteins were collected after 12 000 × *g* for 10 min at 4 °C. The supernatant proteins were separated by 12% SDS-PAGE and transferred onto a polyvinylidene difluoride (PVDF) membrane (Millipore, USA). The membrane was blocked with 5% skim milk at 25 ℃ for 1 h, and then incubated with mouse anti-N monoclonal antibody at a dilution of 1:2500 and anti-actin antibody at a dilution of 1:2500 at 25 ℃ for 1 h. After washing with PBS containing 0.1% Tween (PBST), the membranes were further incubated with the secondary antibody, HRP-conjugated goat anti-mouse IgG, at a dilution of 1:5000 at 25 ℃ for 1 h. The immune-stained proteins were visualized using an ECL chemiluminescent detection system according to the manufacturer’s instructions.

### Real-time quantitative PCR (RT-qPCR)

MARC-145 cells [20] in 12-well plates were infected with rPEDV and rPEDV-E_N13A_ at an MOI of 0.05. After 1 h of adsorption, the supernatants were removed, and the cells were washed with PBS three times and cultured in a maintenance medium. At the indicated time points, the cells were washed with PBS three times and collected for total RNA extraction using RNAiso Plus (Takara, Japan). Then, the reverse transcription was performed using HiScript II Q RT SuperMix for qPCR (Vazyme, China). Amplification was carried out in a 10 μL reaction mixture containing 5 μL ChamQ SYBR® qPCR Master Mix (Vazyme, China), 0.25 μL primers, 2 μL ddH_2_O, and 2.5 μL cDNA. The reaction procedure was 95 °C for 30 s, followed by 40 cycles at 95 °C for 10 s and 60 °C for 30 s. The primers for the detection of cytokines are also listed in Table 1. The relative mRNA expression level was normalized to the housekeeping gene β-actin or GAPDH.

### Growth kinetics

Multistep growth kinetics were performed as before with little modification [19]. MARC-145 cells in 12-well plates were infected with PEDV at an MOI of 0.05. After 1 h absorption, the cells were washed with PBS three times and maintained in a maintenance medium. The virus titers of the supernatants at the indicated time points were determined by the TCID_50_ assay.

### Plaque assay

The plaque assay was performed according to the previous method [19]. MARC-145 cells in 6-well plates were infected with 2 mL of tenfold serially diluted PEDV viruses. After 1 h absorption, the cells were washed with PBS three times and overlaid with 1% low-melting agarose in DMEM. Plaques were visualized by the neutral red dye.

### Luciferase assay for NF-κB activity

HEK-293T cells were co-transfected with NF-κB promoter plasmid, pRL-TK, and pCAGGS-E, pCAGGS-E_N13A_ or pCAGGS vector as negative control. Cells were harvested at 24 h post-transfection and assayed for luciferase activity.

### Evaluation of pathogenicity of the recombinant strains

Twenty-one newborn piglets from the same pigsty in a nearby pig farm of the research institute, with transmissible gastroenteritis virus (TGEV), PEDV, porcine delta coronavirus (PDCoV), and rotavirus (RV) negative, were randomly divided into three groups, with seven piglets in each group. At 2 days of birth, piglets were orally inoculated with a dose of 10^6^ TCID_50_/piglet of the recombinant strains or mock inoculated with equal volume DMEM. The animals were monitored every day for clinical signs of disease. Rectal swabs were collected daily, and viral levels in the rectal swab samples were determined. Rectal swabs were collected daily, and viral RNA shedding copies in the rectal swab samples were determined by AceQ Universal U+Probe Master Mix V2 regent (Vazyme, China) using the primers PEDV-N-F, PEDV-N-R, and the N gene probe in Table 1_._ The severity of diarrhea was scored as previous research: 0, solid; 1, pasty; 2, semiliquid (mild diarrhea); and 3, liquid (severe diarrhea) [21]. Small intestine tissues were fixed in a 4% paraformaldehyde solution and stained with hematoxylin and eosin (H&E) stain. The expression levels of pro-inflammatory cytokines in small intestine were detected by RT-qPCR using the primers in Table 1 as mentioned above.

### Statistical analysis

The statistical analyses were performed using GraphPad Prism 8.0.2. The data were analyzed by a *t*-test or one-way analysis of variance (ANOVA). *P < 0.05, **P < 0.01, and ***P < 0.001 were considered statistically significant. Error bars indicate means ± standard deviations (SD).

## Results

### Homology analysis of the coronavirus E protein

**Figure 1:**
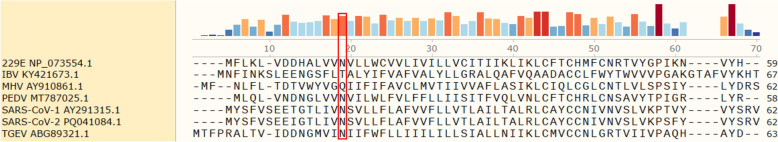
**Multiple sequence alignment of coronavirus E proteins.** The polar uncharged residues are framed in red. The alignment was performed using the SnapGene software. GenBank accession numbers of the E protein sequences are as follows: 229E (NP_073554.1), IBV (KY421673.1), MHV AY910861.1, PEDV (MT787025.1), SARS-CoV-1 (AY291315.1), SARS-CoV-2 (PQ041084.1), TGEV (ABG89321.1).

The 15^th^ polar uncharged residue asparagine of the SARS-CoV-1 E protein plays a key role in inflammasome activation and virulence [22]. To clarify the key amino acid sites of PEDV E protein triggering inflammatory response, a multiple sequence alignment was conducted and the result show that a polar uncharged residue is conserved at position 13 (Figure 1).

### The effect of overexpression of E_N13A_ on the activation of inflammatory response

**Figure 2:**
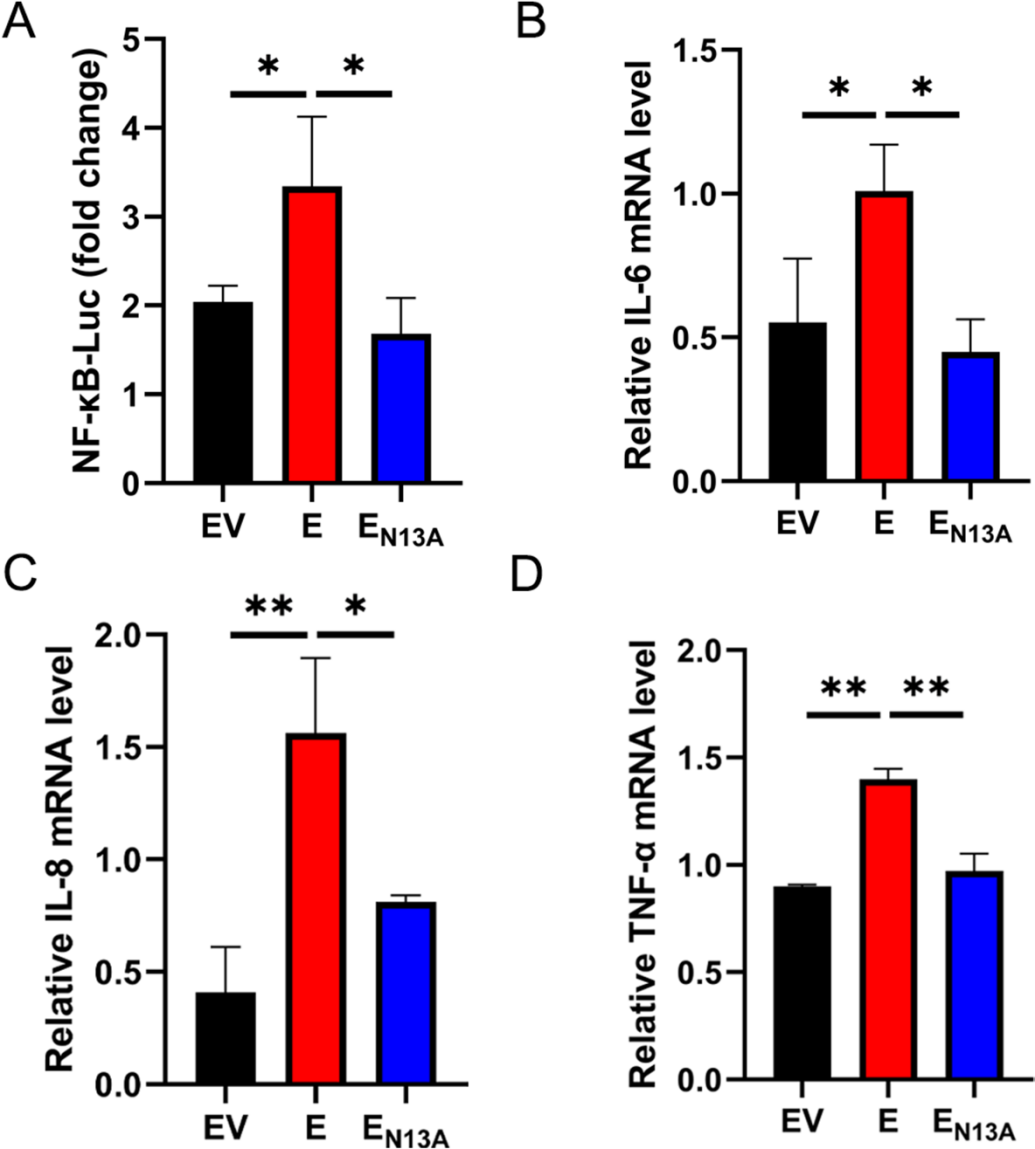
**The effect of overexpression of E**_**N13A**_** on the activation of inflammatory response.** **A** HEK-293T cells were co-transfected with NF-κB promoter plasmid, pRL-TK, and pCAGGS-E, pCAGGS-E_N13A_ or pCAGGS vector as a negative control. Cells were harvested at 24 h post-transfection and assayed for luciferase activity. **B**–**D** HEK-293T cells were transfected with pCAGGS-E, pCAGGS-E_N13A_ or pCAGGS vector as a negative control for 24 h, followed by RT-qPCR to examine the IL-6, IL-8 and TNF-α mRNA level. Error bars indicate standard deviations. The significance level was expressed as *P < 0.05, or **P < 0.01.

The coronavirus E protein is involved in many biological processes [22, 23], and PEDV E can activate the NF-κB pathway and up-regulates the transcriptions of pro-inflammatory cytokines, such as IL-6 and IL-8 [4, 24]. The conserved polar uncharged residue at position 13 was predicted to be a key amino acid for the induction of pro-inflammatory cytokines. To verify this hypothesis, eukaryotic expression plasmids of E and E_N13A_ were successfully constructed. NF-κB pathway plays a key role in regulating the expression of multiple pro-inflammatory cytokines. Hence, we initially determined whether E_N13A_ modulates NF-κB activity by using a luciferase report system. HEK-293T cells were co-transfected with NF-κB-Luc and pCAGGS-E/pCAGGS-E_N13A_, or along with pCAGGS. At 24 h post-transfection, the luciferase activity was detected. As shown in Figures 2A, E significantly enhanced NF-κB promoter activity, but E_N13A_ reduced this activating effect. We further monitored the mRNA level of the cytokines IL-8, TNF-α, and IL-6 after the transfection of pCAGGS-E/pCAGGS-E_N13A_ or pCAGGS. The results indicate that mRNA level of IL-8, TNF-α and IL-6 significantly upregulated the E protein group. Compared with the E protein transfection group, the E_N13A_ group shows significant downregulation of IL-8, IL-6 and TNF-α mRNA levels (Figures 2B–D). These findings indicate that the 13th amino acid of E protein is crucial for its activation of the NF-κB pathway and upregulating inflammatory cytokine expression.

### Design and rescue of a recombinant strain with an N13A mutation in the PEDV E protein

**Figure 3:**
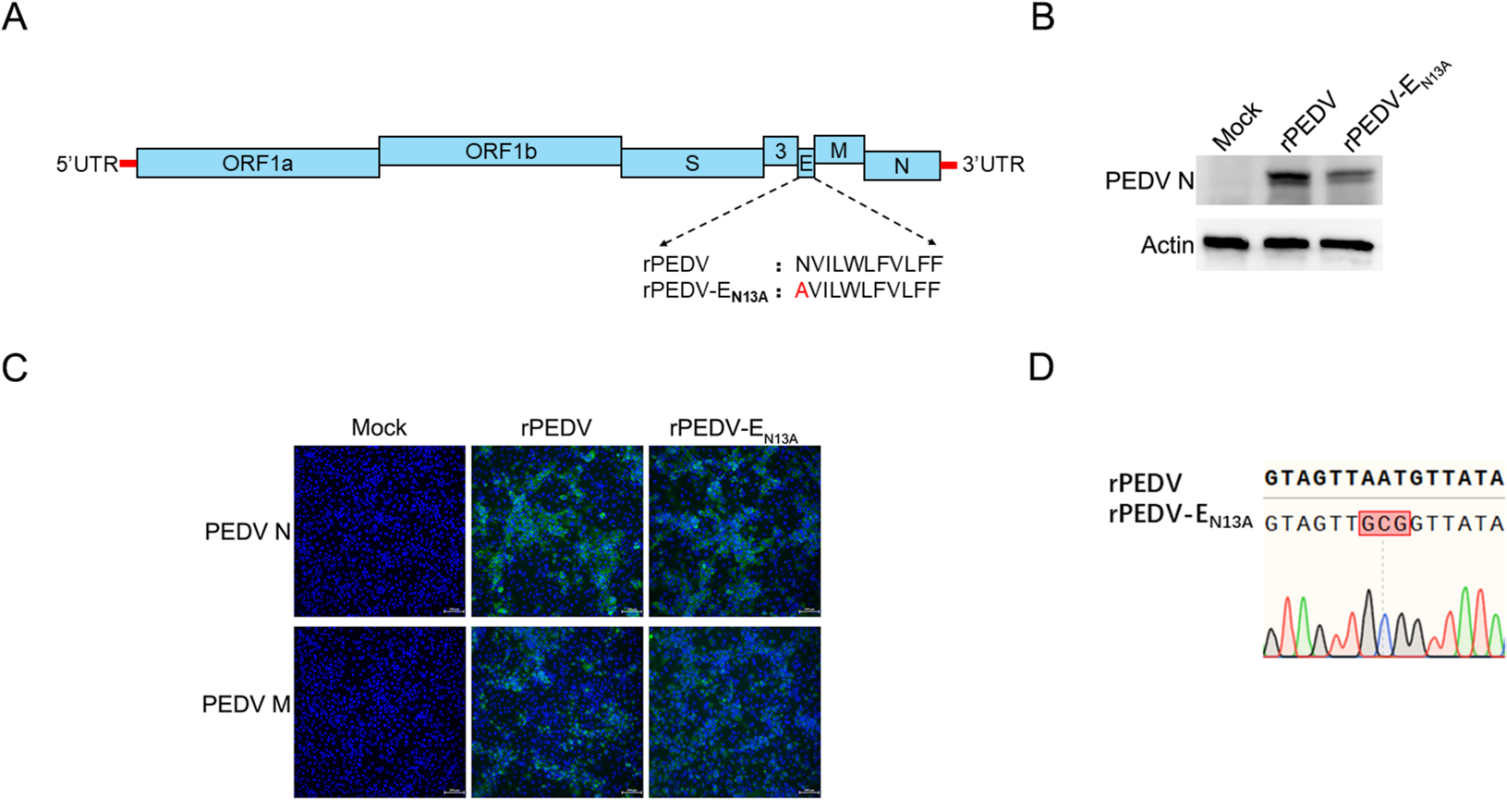
**Rescue of a recombinant strain with the N13A mutation in the PEDV E protein.** **A** Schematic illustration of construction of the rPEDV-E_N13A_ strain. **B** MARC-145 cells were grown on a 12-well plate infected with rPEDV, rPEDV-E_N13A_, or mock for 36 h. Cells were washed with PBS and western blot was performed using N monoclonal antibodies to detect the rescue status of the viruses. **C** MARC-145 cells were grown on a 12-well plate infected with rPEDV, rPEDV-E_N13A_, or mock for 36 h. Cells were washed with PBS, and IFA was performed using N and M monoclonal antibodies to detect the rescue status of the viruses. **D** MARC-145 cells were grown on a 12-well plate infected with rPEDV, rPEDV-E_N13A_, or mock for 36 h. Cells were collected and Sanger sequencing was performed to detect the rescue status of the viruses.

A DNA-launched reverse genetics system based on the bacterial artificial chromosome (BAC) has been successfully developed by our group [19]. The above research indicates that the 13th amino acid of E protein affects its function. To explore the effect of asparagine at position 13 of the E protein on the viral behaviors, we introduced a mutation at position 13 in PEDV E that replaced the asparagine with alanine using the virulent rPEDV as the backbone (Figure 3A). Western blot (Figure 3B) performed by the N monoclonal antibody and IFA (Figure 3C) conducted by the N and M monoclonal antibody indicated that both the rPEDV and rPEDV-E_N13A_ strains were successfully rescued. Sanger sequencing further shows that the rescued recombinant strain was the N13A mutant strain (Figure 3D).

### In vitro characterization of recombinant strains

**Figure 4:**
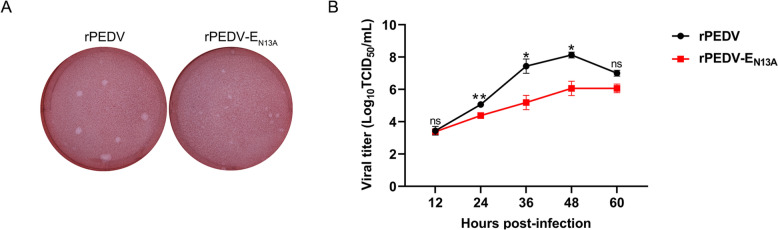
**In vitro characterization of the rPEDV-E**_**N13A**_. **A** MARC-145 cells were infected with rPEDV, rPEDV-E_N13A_, and the plaque morphologies were monitored. **B** MARC-145 cells in 12-well plates were infected with rPEDV and rPEDV-E_N13A_ at an MOI of 0.05. The supernatant was harvested at 12, 24, 36, 48, and 60 hpi and titrated on MARC-145 cells. Error bars indicate standard deviations. The significance level was expressed as *P < 0.05, or **P < 0.01.

To investigate the impact of the N13A mutant on PEDV growth characterization, plaque size, and growth curves in infected MARC-145 cells were monitored. The results indicate that plaques formed by the rPEDV-E_N13A_ strain were smaller than those caused by wild-type PEDV (Figure 4A). The growth kinetics of the rPEDV-E_N13A_ mutant was studied with MARC-145 cells infected at a multiplicity of infection (MOI) of 0.05, and then virus titers in culture supernatants were determined by TCID_50_ assay. The titers of the rPEDV-E_N13A_ mutant at 24, 36, and 48 hpi in the culture supernatant were lower than those corresponding to the parental virus at the same time points (Figure 4B). The data clearly show that the growth of rPEDV-E_N13A_ strain is defective in vitro.

### Recombinant rPEDV-E_N13A_ strain induces significantly lower inflammatory responses in MARC-145 cells and Vero cells

**Figure 5:**
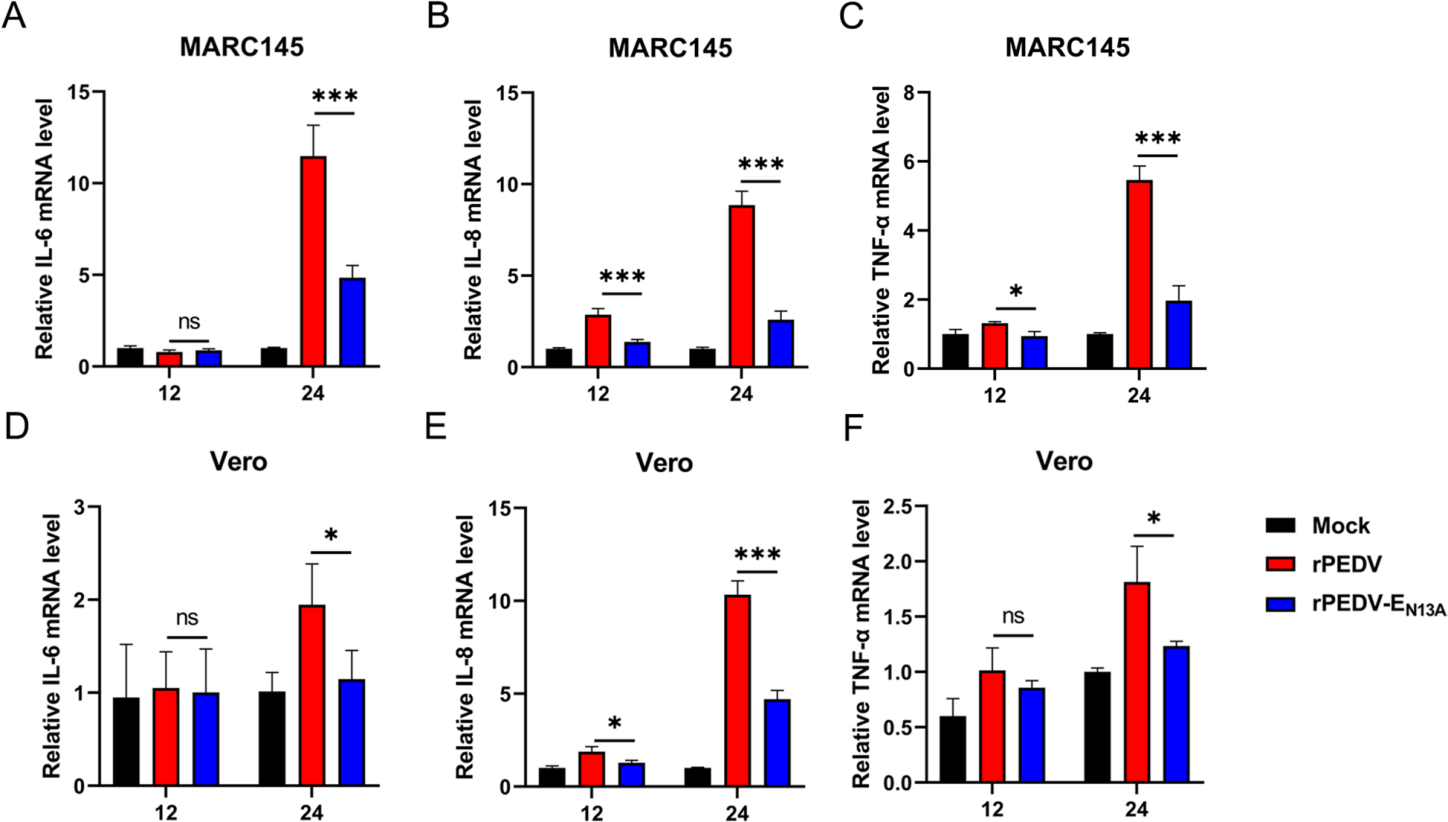
**Recombinant rPEDV-E**_**N13A**_ **strain induces significantly lower inflammatory responses in MARC-145 cells and Vero cells.** MARC-145 cells and Vero cells in 12-well plates were infected with rPEDV and rPEDV- E_N13A_ at an MOI of 0.05. **A**–**G** Cells were collected at 12 and 24 hpi, and RT-qPCR was used to detect the relative expression of the indicated mRNA. Error bars indicate standard deviations. The significance level was expressed as *P < 0.05 or ***P < 0.001.

Based on the coronavirus E protein alignment, the equivalent position in PEDV E is N13. Compared with the E protein, the exogenous expression of E_N13A_ induced lower pro-inflammatory cytokines. To clarify the effect of N13A on the inflammatory response at the whole virus level, MARC-145 cells and Vero cells were infected with rPEDV or rPEDV-E_N13A_ at an MOI of 0.05. At different time points post infection, the mRNA level of the cytokines TNF-α, IL-8, and IL-6 were monitored by RT-qPCR. As shown in Figure 5, significant down-regulation of TNF-α, IL-8, and IL-6 at the transcriptional levels was observed in the rPEDV-E_N13A_ infected group compared to the rPEDV infected group. These findings indicate that N13 plays a crucial role in the inflammatory activation of PEDV E protein at the whole virus level.

### The recombinant strains were attenuated in piglets

**Figure 6:**
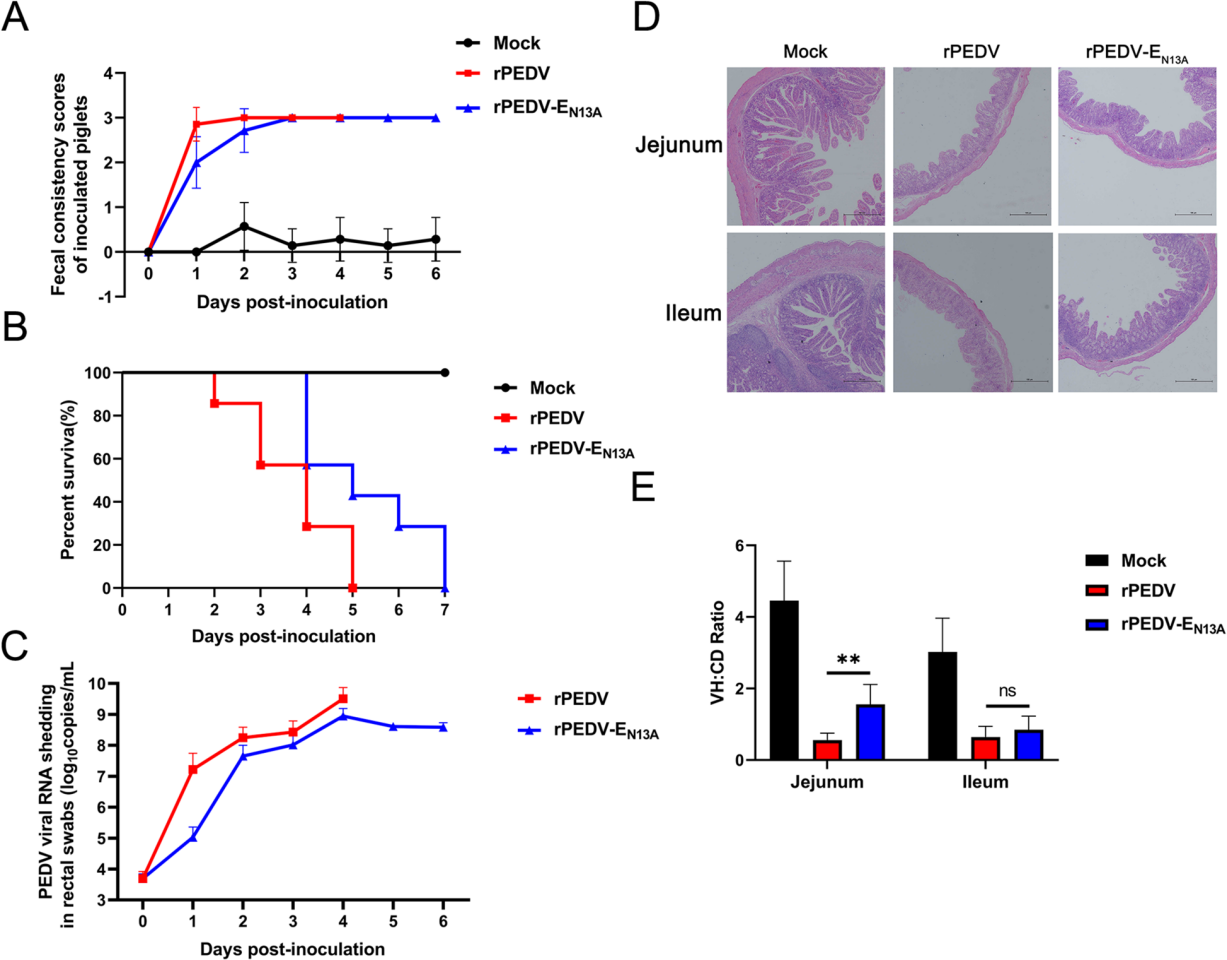
**Pathogenicity of the recombinant strains in piglets.** Piglets were monitored each day after being infected with the recombinant strains. **A** Fecal scores of pigs. Fecal scores were scored as follows: 0, solid; 1, pasty; 2, semiliquid; and 3, liquid. Each line indicates the mean score of a group. **B** Survival curves of piglets. **C** Fecal shedding of PEDV RNA. Viral RNA was isolated from rectal swab samples daily and subjected to RT-qPCR to determine the PEDV N gene RNA copies. **D** H&E staining of the jejunum and ileum from dying or euthanized piglets. **E** VH/CD ratios for piglets. Ten or eleven villi of each intestinal section were measured. Error bars indicate standard deviations. The significance level was expressed as **P < 0.01.

To assess the pathogenicity of the recombinant rPEDV-E_N13A_ strain, 21 newborn piglets were randomly divided into three groups, with seven pigs per group. The piglets were orally inoculated with the rPEDV or rPEDV-E_N13A_ recombinant strains at a dose of 10^6^ TCID_50_ or Mock group with equal volume DMEM. Although the final diarrhea rate in all groups was 100%, the rPEDV-E_N13A_ group reached the peak of diarrhea on the third day after infection, while the rPEDV group reached the peak of diarrhea on the second day (Figure 6A). Furthermore, compared to the rPEDV group, rPEDV-E_N13A_ induced relatively milder diarrhea on the first and second day (Figure 6A). Compared with the parental strain group, the time when piglets in the rPEDV-E_N13A_ group began to die and the time when all piglets had died was delayed by two days (Figure 6B). In addition, compared to the highest level of viral shedding (3.22 × 10^9^ copies/mL) of the rPEDV group, rPEDV-E_N13A_ (8.9 × 10^8^ copies/mL) piglets had lower viral fecal RNA shedding (Figure 6C). Hematoxylin and eosin (H&E) staining revealed rPEDV-E_N13A_ strains caused milder histopathological lesions to intestinal villi compared to the rPEDV in the jejunum (Figure 6D). The villous height/crypt depth (VH/CD) ratios of the jejunum of the rPEDV-E_N13A_ infected pigs were significantly higher than the rPEDV infection groups (Figure 6E). In general, these data indicate that the rPEDV-E_N13A_ recombinant strains were attenuated in neonatal piglets.

### Recombinant rPEDV-E_N13A_ strain induces significantly lower inflammatory responses in vivo

**Figure 7:**
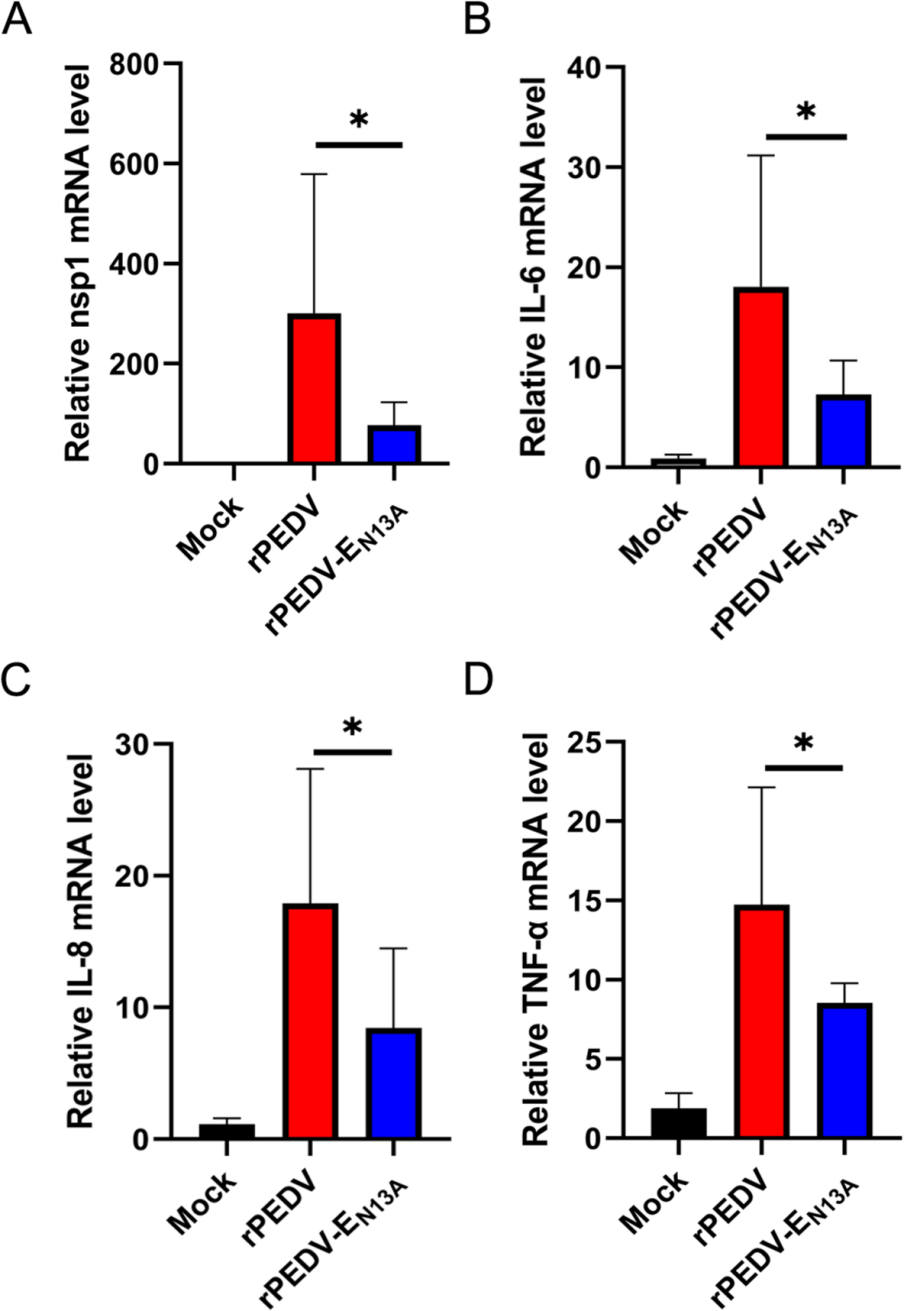
**Recombinant rPEDV-E**_**N13A**_** strain induces significantly lower inflammatory responses in vivo.** **A**–**D** The jejunum of piglets was collected, and RT-qPCR was used to detect the relative expression of the indicated mRNA. Error bars indicate standard deviations. The significance level was expressed as *P < 0.05.

To clarify the effect of N13A on the inflammatory response in vivo. The small intestines of piglets were collected and the levels of inflammatory factors and viruses were detected. Compared to the rPEDV infected group, the viral load in the jejunum of rPEDV-E_N13A_ infected pigs was significantly reduced (Figure 7A). Besides, rPEDV-E_N13A_ induced lower mRNA expression levels of the cytokines IL-6, IL-8, and TNF-α (Figures 7B–D).

## Discussion

Since the 1970s, PEDV has been endemic in many countries. In 2010, highly virulent variant PEDV strains appearing in China have brought huge economic losses to the global pork industry. Currently, PEDV remains a major challenge affecting the healthy development of the pork industry worldwide. Vaccine immunization and medication are effective approaches to control the spread of PEDV. However, the largely unknown pathogenic mechanism of PEDV severely hinders the development of effective vaccines and drugs.

Inflammatory responses occur when the host immune system fights against invading pathogens, which are mediated by multiple chemokines and cytokines. However, excessive inflammatory response can also damage the body. Viral infection usually induces excessive inflammation and leads to further damage to the organism. Inflammation storm is one of the essential factors leading to the aggravation or death of patients with COVID-19 [25]. Many SARS-CoV-2 proteins can activate inflammatory responses. SARS-CoV-2 ORF8 binds to dendritic cells (DC) and induces the secretion of IL-1β, IL-6, IL-12p70, TNF-α, MCP-1, and IL-10 [26]. SARS-CoV-2 E protein induced hyperinflammation by interaction with TMED10 [12]. SARS-CoV-2 NSP16 promotes IL-6 production by regulating the stabilization of HIF-1α [27]. Porcine coronavirus PEDV and PDCoV can also cause the expression of pro-inflammatory cytokine, which is related to the enteritis and tissue damage [28, 29]. Many PEDV proteins have been identified as inflammatory inducers, such as the membrane (M), N, E, and nsp4 proteins [4, 30–32]. However, whether the inflammatory response induced by these proteins is part of PEDV virulence has not yet been elucidated.

Two point mutations in the SARS-CoV-1 E protein N15A and V25F, homologous to the T16A and A26F in IBV (the infectious bronchitis virus, IBV) E protein, are responsible for the activation of inflammatory response and pathogenicity [33]. The two equivalent positions in PEDV E are N13 and F23. The strains with the mutations at the corresponding two sites in SARS-CoV-1 and IBV can be successfully rescued. Surprisingly, although the N13A mutant strain was successfully rescued (Figure 3), the E protein carrying the F23V mutation was unable to be successfully rescued after multiple attempts (data not shown), highlighting the differences between different coronaviruses. In addition, the in vitro growth curves of SARS-CoV-1 and IBV mutants were similar to their parents, while the growth kinetics of PEDV E mutant were significantly lower compared to the parental strain (Figure 4), which further suggests that the equivalent amino acids of PEDV or alpha coronavirus E protein may have more biological functions compared to other coronaviruses.

In conclusion, N13 of E protein has been identified as a key amino acid site responsible for the activation of inflammation. The E_N13A_ protein or the rPEDV-E_N13A_ strain induces lower inflammatory response, and the rPEDV-E_N13A_ strain is attenuated in piglets. Our findings enrich the pathogenic mechanism of PEDV and provide a new target for the development of PEDV vaccines and drugs.

## Data Availability

All the data generated during the current study are included in the manuscript.

## References

[1] Li YC, Wu QX, Huang LL, Yuan C, Wang JL, Yang Q (2018) An alternative pathway of enteric PEDV dissemination from nasal cavity to intestinal mucosa in swine. Nat Commun 9:3811 30232333 10.1038/s41467-018-06056-w PMC6145876

[2] Li YC, Wang XY, Zhang E, Liu RL, Yang CJ, Duan Y, Jiang YQ, Yang Q (2022) Calpain-1: a novel antiviral host factor identified in porcine small intestinal mucus. mBio 13:e0035822 36102516 10.1128/mbio.00358-22 PMC9600339

[3] Zhao YX, Fan BC, Song X, Gao J, Guo RL, Yi C, He ZM, Hu HP, Jiang JH, Zhao LX, Zhong TY, Li B (2024) PEDV-spike-protein-expressing mRNA vaccine protects piglets against PEDV challenge. mBio 15:e0295823 38231557 10.1128/mbio.02958-23 PMC10865985

[4] Wu Y, Wang YR, Wang XP, Li MW, Yan HX, Shi HY, Shi D, Chen JF, Guo LJ, Feng L (2024) Elevation of IL-8 secretion induced by PEDV infection via NF-kappaB signaling pathway. Front Cell Infect Microbiol 14:1422560 39104852 10.3389/fcimb.2024.1422560 PMC11298435

[5] Li ZW, Ma ZQ, Han WG, Chang CZ, Li Y, Guo XY, Zheng ZF, Feng YT, Xu LL, Zheng HX, Wang XL, Xiao SQ (2023) Deletion of a 7-amino-acid region in the porcine epidemic diarrhea virus envelope protein induces higher type I and III interferon responses and results in attenuation in vivo. J Virol 97:e0084723 37681956 10.1128/jvi.00847-23 PMC10537754

[6] Zheng L, Wang XH, Guo DX, Cao JL, Cheng LX, Li XZ, Zou DH, Zhang YT, Xu JX, Wu XN, Shen YJ, Wang HY, Yu W, Li LY, Xiao LJ, Song BF, Ma JZ, Liu XY, Li PF, Xu SY, Xu X, Zhang H, Wu ZJ, Cao HW (2021) Porcine epidemic diarrhea virus E protein suppresses RIG-I signaling-mediated interferon-β production. Vet Microbiol 254:108994 33486326 10.1016/j.vetmic.2021.108994

[7] Hoytema van Konijnenburg DP, Nigrovic PA, Zanoni I (2024) Regional specialization within the mammalian respiratory immune system. Trends Immunol 45:871–891 39438172 10.1016/j.it.2024.09.011 PMC11560516

[8] Zhang XH, Chen GY, Yin JQ, Nie LC, Li LH, Du Q, Tong DW, Huang Y (2024) Pseudorabies virus UL4 protein promotes the ASC-dependent inflammasome activation and pyroptosis to exacerbate inflammation. PLoS Pathog 20:e1012546 39316625 10.1371/journal.ppat.1012546 PMC11421794

[9] Kucia M, Ratajczak J, Bujko K, Adamiak M, Ciechanowicz A, Chumak V, Brzezniakiewicz-Janus K, Ratajczak MZ (2021) An evidence that SARS-Cov-2/COVID-19 spike protein (SP) damages hematopoietic stem/progenitor cells in the mechanism of pyroptosis in NLRP3 inflammasome-dependent manner. Leukemia 35:3026–3029 34163002 10.1038/s41375-021-01332-z PMC8219510

[10] Malireddi RKS, Sharma BR, Kanneganti TD (2024) Innate immunity in protection and pathogenesis during coronavirus infections and COVID-19. Annu Rev Immunol 42:615–645 38941608 10.1146/annurev-immunol-083122-043545 PMC11373870

[11] De Sa KSG, Amaral LA, Rodrigues TS, Caetano CCS, Becerra A, Batah SS, Lopes FT, de Oliveira IM, Lopes LS, Almeida L, Mota CM, Oliveira S, Wada DT, Koenigkam-Santos M, Martins RB, Rosales RRC, Arruda E, Fabro AT, Zamboni DS (2024) Pulmonary inflammation and viral replication define distinct clinical outcomes in fatal cases of COVID-19. PLoS Pathog 20:e1012222 38838044 10.1371/journal.ppat.1012222 PMC11182505

[12] Liu L, Zhang LJY, Hao XY, Wang Y, Zhang XC, Ge L, Wang PH, Tian BX, Zhang M (2024) Coronavirus envelope protein activates TMED10-mediated unconventional secretion of inflammatory factors. Nat Commun 15:8708 39379362 10.1038/s41467-024-52818-0 PMC11461611

[13] Liang SX, Bao CL, Yang Z, Liu SY, Sun YN, Cao WT, Wang T, Schwantes-An TH, Choy JS, Naidu S, Luo A, Yin WG, Black SM, Wang J, Ran PX, Desai AA, Tang HY (2023) SARS-CoV-2 spike protein induces IL-18-mediated cardiopulmonary inflammation via reduced mitophagy. Signal Transduct Target Ther 8:108 36894537 10.1038/s41392-023-01368-w PMC9998025

[14] Pan P, Shen MM, Yu ZY, Ge WW, Chen KL, Tian MF, Xiao F, Wang ZW, Wang J, Jia YL, Wang WB, Wan P, Zhang J, Chen WJ, Lei ZW, Chen X, Luo Z, Zhang QW, Xu M, Li G, Li YK, Wu JG (2021) SARS-CoV-2 N protein promotes NLRP3 inflammasome activation to induce hyperinflammation. Nat Commun 12:4664 34341353 10.1038/s41467-021-25015-6 PMC8329225

[15] Zhang Y, Chen HJ, Yu J, Feng R, Chen Z, Zhang XL, Ren YD, Yang GJ, Huang XD, Li GX (2022) Comparative transcriptomic analysis of porcine epidemic diarrhea virus epidemic and classical strains in IPEC-J2 cells. Vet Microbiol 273:109540 35987184 10.1016/j.vetmic.2022.109540

[16] Niu XY, Kong FZ, Xu JY, Liu MD, Wang QH (2022) Mutations in porcine epidemic diarrhea virus nsp1 cause increased viral sensitivity to host interferon responses and attenuation in vivo. J Virol 96:e0046922 35583324 10.1128/jvi.00469-22 PMC9175621

[17] Peng Q, Fan BC, Song X, He WL, Wang CH, Zhao YX, Guo WL, Zhang X, Liu SY, Gao J, Li KM, Zhang BT, Zhou JZ, Li YC, Guo RL, Li B (2023) Genetic signatures associated with the virulence of porcine epidemic diarrhea virus AH2012/12. J Virol 97:e0106323 37732788 10.1128/jvi.01063-23 PMC10617547

[18] Zheng ZF, Ling X, Li Y, Qiao S, Zhang SQ, Wu J, Ma ZQ, Li MY, Guo XY, Li ZW, Feng YT, Liu X, Goodfellow IG, Zheng HX, Xiao SQ (2024) Host cells reprogram lipid droplet synthesis through YY1 to resist PRRSV infection. mBio 15:e0154924 38953350 10.1128/mbio.01549-24 PMC11323570

[19] Li ZW, Ma ZQ, Dong LF, Yang T, Li Y, Jiao D, Han WG, Zheng HX, Xiao SQ (2022) Molecular mechanism of porcine epidemic diarrhea virus cell tropism. mBio 13:e0373921 35285698 10.1128/mbio.03739-21 PMC9040822

[20] Feng YT, Guo XY, Tian H, He Y, Li Y, Jiang XL, Zheng HX, Xiao SQ (2021) Induction of HOXA3 by PRRSV inhibits IFN-I response through negatively regulation of HO-1 transcription. J Virol 96:e0186321 34851144 10.1128/JVI.01863-21 PMC8827019

[21] Hou YX, Ke HZ, Kim J, Yoo D, Su YF, Boley P, Chepngeno J, Vlasova AN, Saif LJ, Wang QH (2019) Engineering a live attenuated porcine epidemic diarrhea virus vaccine candidate via inactivation of the viral 2’-*O*-Methyltransferase and the endocytosis signal of the spike protein. J Virol 93:e00406-19 31118255 10.1128/JVI.00406-19 PMC6639265

[22] Nieto-Torres JL, DeDiego ML, Verdia-Baguena C, Jimenez-Guardeno JM, Regla-Nava JA, Fernandez-Delgado R, Castano-Rodriguez C, Alcaraz A, Torres J, Aguilella VM, Enjuanes L (2014) Severe acute respiratory syndrome coronavirus envelope protein ion channel activity promotes virus fitness and pathogenesis. PLoS Pathog 10:e1004077 24788150 10.1371/journal.ppat.1004077 PMC4006877

[23] Wang ZH, Qiu MM, Ji Y, Chai KL, Liu CX, Xu FW, Guo F, Tan J, Liu RK, Qiao WT (2024) Palmitoylation of SARS-CoV-2 envelope protein is central to virus particle formation. J Virol 98:e0107224 39287388 10.1128/jvi.01072-24 PMC11495019

[24] Sun M, Ma JL, Yu ZYQ, Pan ZH, Lu CP, Yao HC (2017) Identification of two mutation sites in spike and envelope proteins mediating optimal cellular infection of porcine epidemic diarrhea virus from different pathways. Vet Res 48:44 28854955 10.1186/s13567-017-0449-y PMC5577753

[25] Asrani P, Hassan MI (2021) SARS-CoV-2 mediated lung inflammatory responses in host: targeting the cytokine storm for therapeutic interventions. Mol Cell Biochem 476:675–687 33064288 10.1007/s11010-020-03935-z PMC7563911

[26] Hamdorf M, Imhof T, Bailey-Elkin B, Betz J, Theobald SJ, Simonis A, Di Cristanziano V, Gieselmann L, Dewald F, Lehmann C, Augustin M, Klein F, Alejandre Alcazar MA, Rongisch R, Fabri M, Rybniker J, Goebel H, Stetefeld J, Brachvogel B, Cursiefen C, Koch M, Bock F (2024) The unique ORF8 protein from SARS-CoV-2 binds to human dendritic cells and induces a hyper-inflammatory cytokine storm. J Mol Cell Biol 15:mjad062 37891014 10.1093/jmcb/mjad062 PMC11181941

[27] Mou XL, Luo F, Zhang WH, Cheng Q, Hepojoki J, Zhu SW, Liu YY, Xiong HR, Guo DY, Yu JY, Chen LJ, Li YR, Hou W, Chen SL (2024) SARS-CoV-2 NSP16 promotes IL-6 production by regulating the stabilization of HIF-1alpha. Cell Signal 124:111387 39251053 10.1016/j.cellsig.2024.111387

[28] Zhang HL, Han FF, Shu XL, Li QQ, Ding QW, Hao CL, Yan XG, Xu ML, Hu H (2022) Co-infection of porcine epidemic diarrhoea virus and porcine deltacoronavirus enhances the disease severity in piglets. Transbound Emerg Dis 69:1715–1726 33960702 10.1111/tbed.14144

[29] Guo XY, Feng YT, Zhao XJ, Qiao S, Ma ZQ, Li ZW, Zheng HX, Xiao SQ (2023) Coronavirus porcine epidemic diarrhea virus utilizes chemokine interleukin-8 to facilitate viral replication by regulating Ca(2+) flux. J Virol 97:e0029223 37133374 10.1128/jvi.00292-23 PMC10231212

[30] Yu LY, Dong JG, Wang YW, Zhang PF, Liu YL, Zhang LY, Liang PS, Wang L, Song CX (2019) Porcine epidemic diarrhea virus nsp4 induces pro-inflammatory cytokine and chemokine expression inhibiting viral replication in vitro. Arch Virol 164:1147–1157 30799511 10.1007/s00705-019-04176-2

[31] Xu XG, Zhang HL, Zhang Q, Huang Y, Dong J, Liang YB, Liu HJ, Tong DW (2013) Porcine epidemic diarrhea virus N protein prolongs S-phase cell cycle, induces endoplasmic reticulum stress, and up-regulates interleukin-8 expression. Vet Microbiol 164:212–221 23562137 10.1016/j.vetmic.2013.01.034 PMC7117426

[32] Xu XG, Zhang HL, Zhang Q, Dong J, Huang Y, Tong DW (2015) Porcine epidemic diarrhea virus M protein blocks cell cycle progression at S-phase and its subcellular localization in the porcine intestinal epithelial cells. Acta Virol 59:265–275 26435150 10.4149/av_2015_03_265

[33] Li SM, Yuan LX, Dai G, Chen RA, Liu DX, Fung TS (2019) Regulation of the ER stress response by the ion channel activity of the infectious bronchitis coronavirus envelope protein modulates virion release, apoptosis, viral fitness, and pathogenesis. Front Microbiol 10:3022 32038520 10.3389/fmicb.2019.03022 PMC6992538

[34] Su CM, Kim J, Tang J, Hung YF, Zuckermann FA, Husmann R, Roady P, Kim J, Lee YM, Yoo D (2024) A clinically attenuated double-mutant of porcine reproductive and respiratory syndrome virus-2 that does not prompt overexpression of proinflammatory cytokines during co-infection with a secondary pathogen. PLoS Pathog 20:e1012128 38547254 10.1371/journal.ppat.1012128 PMC11003694

[35] Luo XG, Chen XX, Qiao SL, Li R, Xie S, Zhou XY, Deng RG, Zhou EM, Zhang GP (2020) Porcine reproductive and respiratory syndrome virus enhances self-replication via AP-1-dependent induction of SOCS1. J Immunol 204:394–407 31826939 10.4049/jimmunol.1900731 PMC6943376

[36] Villacampa A, Alfaro E, Morales C, Diaz-Garcia E, Lopez-Fernandez C, Bartha JL, Lopez-Sanchez F, Lorenzo O, Moncada S, Sanchez-Ferrer CF, Garcia-Rio F, Cubillos-Zapata C, Peiro C (2024) SARS-CoV-2 S protein activates NLRP3 inflammasome and deregulates coagulation factors in endothelial and immune cells. Cell Commun Signal 22:38 38225643 10.1186/s12964-023-01397-6 PMC10788971

[37] Pantazi I, Al-Qahtani AA, Alhamlan FS, Alothaid H, Matou-Nasri S, Sourvinos G, Vergadi E, Tsatsanis C (2021) SARS-CoV-2/ACE2 interaction suppresses IRAK-M expression and promotes pro-inflammatory cytokine production in macrophages. Front Immunol 12:683800 34248968 10.3389/fimmu.2021.683800 PMC8261299

[38] Ling XY, Cao ZG, Sun PP, Zhang H, Sun YG, Zhong J, Yin W, Fan KH, Zheng XZ, Li HQ, Sun N (2023) Target discovery of matrine against PRRSV in Marc-145 cells via activity-based protein profiling. Int J Mol Sci 24:11526 37511286 10.3390/ijms241411526 PMC10381006

[39] Jing HY, Fang LR, Wang D, Ding Z, Luo R, Chen HC, Xiao SB (2014) Porcine reproductive and respiratory syndrome virus infection activates NOD2-RIP2 signal pathway in MARC-145 cells. Virology 458–459:162–171 24928048 10.1016/j.virol.2014.04.031

[40] Zhang QZ, Shi KC, Yoo DW (2016) Suppression of type I interferon production by porcine epidemic diarrhea virus and degradation of CREB-binding protein by nsp1. Virology 489:252–268 26773386 10.1016/j.virol.2015.12.010 PMC7111358

[41] Deng XF, Buckley AC, Pillatzki A, Lager KM, Faaberg KS, Baker SC (2020) Inactivating three interferon antagonists attenuates pathogenesis of an enteric coronavirus. J Virol 94:e00565-20 32554697 10.1128/JVI.00565-20 PMC7431798

